# Isolation and Characterization of Squamous Cell Carcinoma-Derived Stem-like Cells: Role in Tumor Formation

**DOI:** 10.3390/ijms141019540

**Published:** 2013-09-26

**Authors:** Katiuscia Dallaglio, Tiziana Petrachi, Alessandra Marconi, Francesca Truzzi, Roberta Lotti, Annalisa Saltari, Paolo Morandi, Mario Puviani, Antonino Maiorana, Dennis R. Roop, Carlo Pincelli

**Affiliations:** 1 Department of Dermatology, University of Modena and Reggio Emilia, Via del Pozzo 71, 41124 Modena, Italy; E-Mails: katiuscia.dallaglio@yahoo.it (K.D.); tiziana.petrachi@virgilio.it (T.P.); alessandra.marconi@unimore.it (A.M.); truzzi.francesca@gmail.com (F.T.); 50329@studenti.unimore.it (R.L.); annalisa.saltari@unimore.it (A.S.); paolo.morandi@unimore.it (P.M.); 2 Ospedale Civile di Sassuolo, Via Francesco Ruini, 2, 41049 Sassuolo (MO), Italy; E-Mail: mar.puviani@ospedalesassuolo.it; 3 Department of Laboratories and Pathologic Anatomy, University of Modena and Reggio Emilia, Via del Pozzo 71, 41124 Modena, Italy; E-Mail: antonino.maiorana@unimore.it; 4 Charles C. Gates Center for Regenerative Medicine and Stem Cell Biology, Department of Dermatology, University of Colorado, Denver, CO 80045, USA; E-Mail: dennis.roop@ucdenver.edu

**Keywords:** squamous cell carcinoma, stem cells, β_1_-integrin, tumor formation, survivin, rapidly adhering cells, skin, tumorigenesis, differentiation

## Abstract

In human epidermis, keratinocyte stem cells (KSC) are characterized by high levels of β_1_-integrin, resulting in the rapid adhesion to type IV collagen. Since epithelial tumors originate from KSC, we evaluated the features of rapidly adhering (RAD) keratinocytes derived from primary human squamous cell carcinoma of the skin (cSCC). RAD cells expressed higher levels of survivin, a KSC marker, as compared to non-rapidly adhering (NRAD) cells. Moreover, RAD cells proliferated to a greater extent and were more efficient in forming colonies than NRAD cells. RAD cells also migrated significantly better than NRAD cells. When seeded in a silicone chamber and grafted onto the back skin of NOD SCID mice, RAD cells formed tumors 2–4 fold bigger than those derived from NRAD cells. In tumors derived from RAD cells, the mitotic index was significantly higher than in those derived from NRAD cells, while Ki-67 and survivin expression were more pronounced in RAD tumors. This study suggests that SCC RAD stem cells play a critical role in the formation and development of epithelial tumors.

## 1. Introduction

Cutaneous squamous cell carcinoma (cSCC) is one of the most frequent skin cancers, second only to basal cell carcinoma (BCC) [1]. Although surgical removal of the lesion is the gold standard for cSCC management, often leading to complete eradication of the tumor, about 8% of cSCC patients undergo relapse and/or metastatic spread of the disease after treatment, suggesting that further studies on the mechanisms underlying cSCCs formation are needed.

cSCCs develop following transformation of normal keratinocytes. Experiments based on chemical carcinogen retention show that only long-lived, slow cycling, non-differentiating keratinocytes are able to retain carcinogens, thus being at the origin of skin cancer formation [2]. Thus, keratinocyte stem cells (KSC), that possess all of these features, are believed to be the “SCC starting cells”, as they accumulate oncogenic mutations and cause tumor formation. This has been recently confirmed in mice expressing oncogenic KRas^G12D^ and carrying a p53 deletion, in which both hair follicle bulge stem cells and their related progeny, but not matrix hair follicle transit amplifying cells, generated invasive SCC of the skin [3]. Although a definite marker for KSC is still lacking, a number of molecules have been used to isolate and identify these cells, including β_1_-integrin [4], and the membrane co-factor protein CD46, which sustains β_1_-integrin-mediated KSC adhesion [5], α6-integrin/CD71 [6], the anti-apoptotic protein survivin [7], and many others. We have previously shown that survivin identifies KSC and is downregulated when β_1_-integrin signal is disrupted [7].

The hypothesis of tumors originating from stem cells has included two main concepts: on one side, tumors can develop from normal stem cells undergoing malignant transformation; on the other side, once tumors are formed, cells retaining stem cells features sustain their growth and are responsible for tumor recurrence and in some cases, distant metastasis [8,9]. The latter cells, named cancer stem cells (CSC), have been initially found in leukemia [10], and later in many solid tumors, including cSCC [11,12]. Recently, multiple populations of CSC in cSCC, characterized by differential expression of CD34 and β_1_-integrin, have been isolated. These cells are controlled by TGF-β and integrin/FAK signaling pathways [13]. In line with these findings, FAK, a tyrosine kinase downstream of integrin signaling, cooperates with β-catenin/Wnt pathway in regulating KSC behavior during early steps of skin carcinogenesis [14]. While these works highlight the importance of integrin signaling in skin tumor formation, recent findings also point at integrins as key players in human cSCC metastasis and invasion, acting through inhibition of apoptosis and differentiation and finally leading to expansion of tumor cells endowed with stem cells properties [15].

In this work, we wanted to isolate from cSCC a subpopulation of primary keratinocytes with stem cell features, based on their high β_1_-integrin levels. We characterized these cells both *in vitro* and *in vivo*, with particular regard to their tumorigenic ability. Keratinocytes expressing high levels of β1-integrin and rapidly adhering to collagen IV (RAD) migrated to a greater extent, displayed higher colony forming efficiency and increased expression of stem cell-associated genes, as compared to non rapidly adhering (NRAD) cells. Moreover, when injected into immune-compromised mice, RAD cells generated bigger and more aggressive tumors, as compared to NRAD and bulk cells.

## 2. Results and Discussion

β1 integrin levels in mice serve as a discriminator between cSCC cells with different tumor-initiating potential [13]. While β1 integrin was used as a marker to enrich highly clonogenic SCC cells *in vitro* by selecting a population of rapidly adhering keratinocytes [16], a study providing partial characterization of SCC subpopulations did not address tumor initiation ability *in vivo* [17]. In the present study, we further enriched a population of rapidly adhering cells from cSCC primary cultures by improving the rapid adhesion to collagen IV method. The isolated subpopulations were then characterized both *in vitro* and *in vivo*, with particular regard to their tumorigenic ability.

### 2.1. RAD Cells from Primary cSCC Cultures Are Abundant and Express High Levels of β_1_-Integrin

Cultured cSCC cells display defective differentiation as compared to normal keratinocytes, and require feeder layers to be abundantly selected *in vitro* [18]. Once cultured for a few passages, cSCC cells become feeder-independent, yet are able to recapitulate tumor heterogeneity when inoculated *in vivo*, thus representing an excellent model for cSCC studies [19]. We therefore used primary cSCCs-derived cell cultures to analyze a population of stem-like cells in this tumor.

Rapid adhesion to collagen-IV, a well-established method to rapidly and easily enrich KSC in human epidermis [4], has been recently applied to cSCC [16]. In the latter study, 20 min of adhesion to collagen IV was used to select a population enriched in highly clonogenic cells from SCC cell lines [16]. By reducing the adhesion period from 20 to 5 min, we were able to obtain a subpopulation further enriched in KSC from normal human skin [unpublished data]. We therefore used the 5-min method to isolate and characterize a population of rapidly adhering SCC keratinocytes from primary cSCC-derived cell cultures.

**Figure 1:**
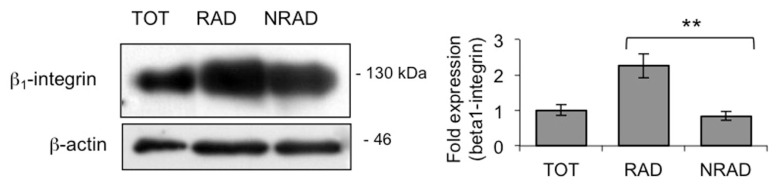
β_1_-integrin levels in cSCC subpopulations. β_1_-integrin levels in RAD, NRAD and TOT cells were analyzed immediately after separation by Western blot. β-actin was used as loading control. Graph shows the average densitometry values normalized to actin, ** *p* < 0.01.

We first observed that RAD/NRAD ratio was 0.45, when isolated from SCC at early passages. RAD cells expressed higher levels of β_1_-integrin than NRAD cells (Figure 1) consistently with high β_1_-integrin expression in cSCCs *in vivo* [20], thus confirming that shortening the adhesion time to collagen IV still allows efficient separation of cells. Interestingly, NRAD cells still display relatively high amount of β_1_-integrin, probably reflecting its overexpression in cSCCs cells when propagated in culture, as previously suggested [21]. At any rate, the choice to characterize SCC cell subtypes immediately after isolation prevents protein expression changes occurring in cell cultures.

### 2.2. RAD from cSCC Are Highly Proliferating Cells *In Vitro*

**Figure 2:**
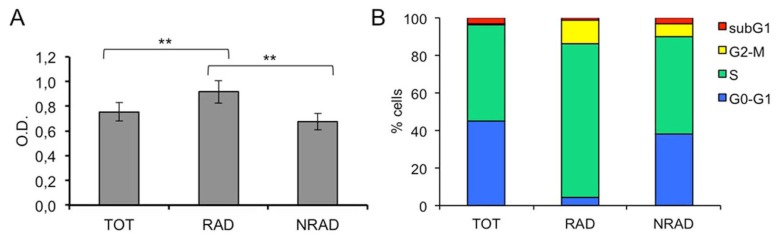

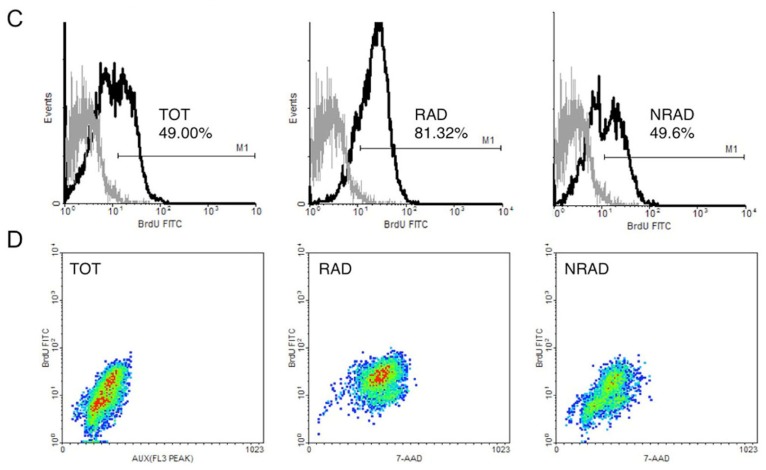
Proliferative ability of cSCCs subpopulations *in vitro*. (**A**) RAD, NRAD and TOT cells ability to proliferate *in vitro* was evaluated by CV staining; (**B**) RAD, NRAD and TOT cells were cultured for 72 h. BrdU incorporation was then evaluated by using FITC BrdU Flow Kit and analyzed by flow cytometry 72 h after the seeding. **p < 0.01; (**C**) Monoparametric histograms showing BrdU incorporation by FACS; (**D**) Density dot plots showing BrdU incorporation by FACS.

β_1_-integrin overexpressing cells from head and neck SCC cell lines, including cSCC, have higher clonogenic ability *in vitro* than cells with low β_1_-integrin levels [16]. In order to analyze the proliferative ability of cSCC subpopulations, we performed a crystal violet (CV) staining of RAD, NRAD and total cell cultures. Proliferation was significantly higher in RAD than in NRAD and total cells (Figure 2A). Stem cells are quiescent *in vivo* under homeostatic conditions, albeit retaining the ability to exit the quiescent state to repopulate and differentiate when necessary. When cultured, stem cells rapidly break the quiescence state and start to proliferate [22]. Consistent with CV assay, BrdU incorporation, an accurate determination of cells in S-phase of the cell cycle by flow cytometry, was higher in RAD than in NRAD and total cells (Figure 2B–D). These data confirm the highest proliferative activity of RAD cells in cSCC *in vitro*.

### 2.3. RAD cSCC Cells Are Less Differentiated and Express High Levels of Survivin

**Figure 3:**
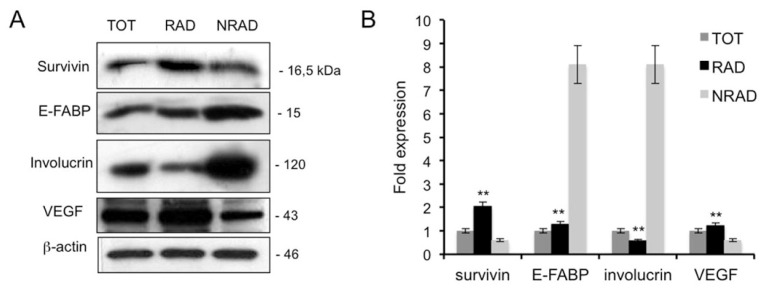
Expression of stem cell and differentiation markers in RAD, NRAD and TOT cells from cSCC. (**A**) Cells were analyzed immediately after separation, and levels of markers were determined by Western blot analysis. β-actin was used as loading control; (**B**) Bar graphs show the average densitometry values normalized to actin. *p < 0.05; **p < 0.01.

Stem cells are undifferentiated cells that give raise to a progeny of transit amplifying cells, which in turn undergo terminal differentiation after a few rounds of division [23]. To further characterize RAD cells, we evaluated the expression of several epidermal differentiation markers in cSCC subpopulations (Figure 3A,B). E-FABP and involucrin were less expressed in RAD than in NRAD cells. Similarly to involucrin, E-FABP is expressed in terminally differentiated keratinocytes and induces differentiation in normal and psoriatic cells *in vitro* [24]. In SCCs, both involucrin and E-FABP mark differentiated keratinocytes [25]. Therefore, overexpression of these markers in NRAD cells suggests that NRAD are highly differentiated cells, while RAD keratinocytes represent a less differentiated subpopulation in the tumor. On the other hand, survivin, a marker of normal KSC *in vitro*, was overexpressed in RAD keratinocytes when compared to NRAD and total cells. Survivin overexpression has been reported in tumors, in fetal tissues and recently in stem cells from adult tissues, in both normal and pathologic conditions [26,27]. Consistently, survivin is involved in the CSC resistance mechanisms against chemo- and radio-therapy [28]. Moreover, regulation of survivin expression decides the balance between survival and apoptosis in neural stem cells and glioma CSC [29]. Finally, vascular endothelial growth factor (VEGF) is slightly but significantly increased in RAD cells, in line with the recent observation of this factor being upregulated in CSC [30]. Taken together, these data suggest that RAD cells represent a population of stem-like cells in primary cultures of cSCC.

### 2.4. RAD Cells from cSCC Display High Colony Forming Efficiency and Increased Expression of Stem Cell-Associated Genes

**Figure 4:**
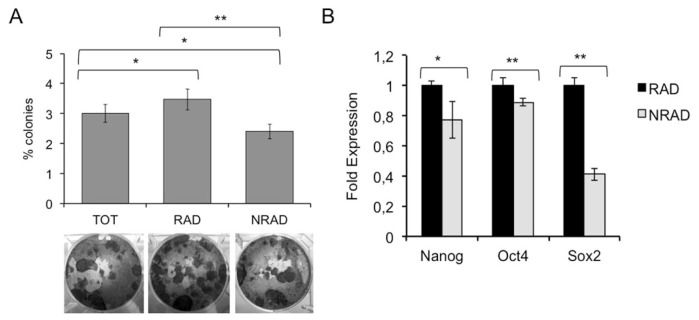
Analysis of stem-cell features in RAD and NRAD cells *in vitro*. (**A**) Clonal growth assessment of cSCC subpopulations by CFE. CFE was performed in triplicate in three independent experiments and quantification is shown in the upper panel. At the bottom, representative pictures of CFE obtained by growing cells at clonal density and stained with CV are shown; (**B**) mRNA expression of Nanog, Oct4 and Sox-2 by Real Time PCR. The different levels of gene expression in RAD *vs.* NRAD cells are shown. *p < 0.05; **p < 0.01.

Colony forming efficiency (CFE) assay assesses the capability of cells to generate progeny. It has been employed to evaluate clonogenic ability of cancer cell subtypes and as a surrogate to analyze putative enrichments of stem cell-like cells [16,31]. CFE analysis of cells re-plated at clonal density immediately after separation showed that RAD cells have a significantly higher CFE as compared to NRAD and total cells (Figure 4A). This is in line with the highest total cell output and proliferation observed in RAD cells, as shown in Figure 2. In addition, the stem cell markers Nanog, Oct4 and Sox-2 were significantly more expressed in RAD cells, further confirming the stem cell nature of these cSCC cells (Figure 4B). Interestingly, the transcription factor Sox-2 directly increases survivin levels in neural stem cells [26], suggesting that it may contribute to sustain RAD stemness also by upregulating survivin in cSCC cells.

CD133 has been successfully used to identify and separate CSC in primary human cSCC [32]. We therefore evaluated CD133 expression in cultured cSCC cells by flow cytometry and Western blot. While Head and Neck SCC cell lines express variable levels of this marker in almost 100% of the cells, primary cSCC cultures do not express detectable levels of this protein (data not shown). CD133 is a very sensitive antigen that may be altered and not detectable in culture [33].

### 2.5. RAD Cells Migrate to a Greater Extent than NRAD and Total SCC Keratinocytes

**Figure 5:**
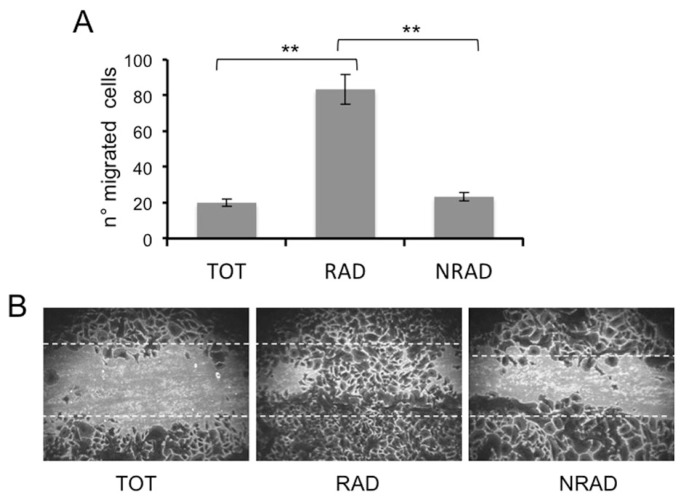
Migration ability of cSCCs subpopulations. The migration of RAD, NRAD and TOT cells was determined by scratching assay. Cells were fixed and stained, and the number of migrated cells to the scratched surface area was counted. (**A**) Number of migrated cells expressed as the mean + SD from triplicate experiments, *p < 0.05, **p < 0.01; (**B**) Representative images of migrated cSCCs subpopulations in the scratching assay.

Along with tumorigenic ability, CSC or cells with stem-cell features may also retain the capacity to migrate and generate distant metastasis. Unlike most BCCs, cSCCs display a high risk to metastasize. Since the ability of cells to form metastasis reflects increased cell motility, we assessed cell migration through a scratching assay. To rule out the possibility that migrated cells were indeed proliferating keratinocytes, cells were pretreated with the mitosis inhibitor mitomycin C. RAD cells possess a higher migration ability than NRAD and total cells (Figure 5A,B). As β_1_-integrin and survivin are highly expressed in RAD cells and are involved in keratinocyte migration [34], [unpublished results], they might sustain this RAD cell ability.

### 2.6. RAD Cells from cSCCs Are Tumorigenic *In Vivo*

**Figure 6:**
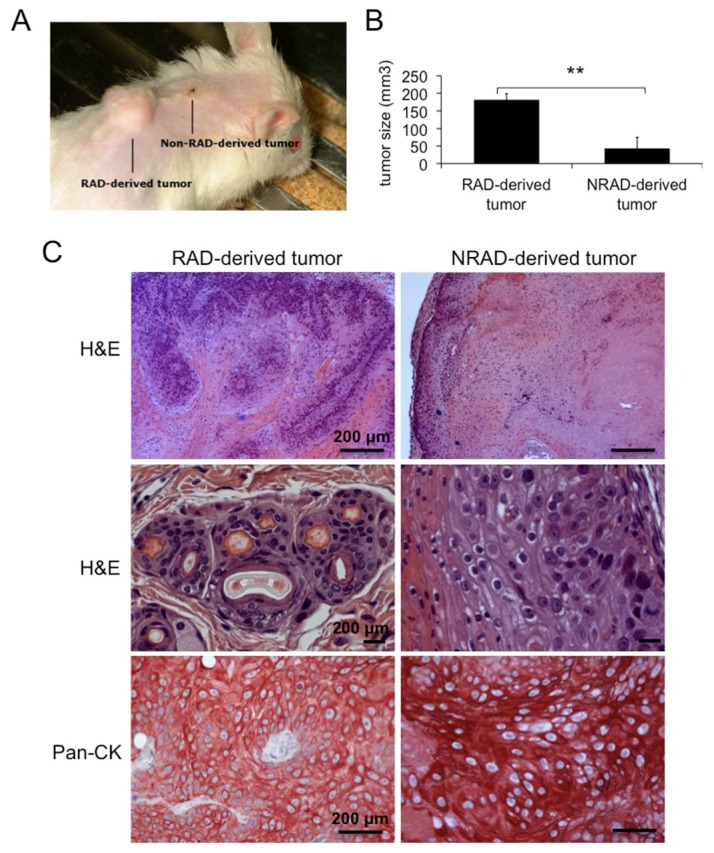
Tumorigenic ability of cSCC cells subpopulations *in vivo.* (**A**) Representative picture of RAD and NRAD-derived tumors formed by xenografting cSCC keratinocytes onto NOD/SCID mice; (**B**) Size of RAD and NRAD derived tumors as measured in three independent experiments. *p < 0.05, **p < 0.01; (**C**) Hematoxilin & Eosin and Pan-cytokeratin (CK) staining of RAD and NRAD-derived tumors. Scale bars = 200 μm.

Although “rapid adhesion to collagen IV” is a useful method to isolate cSCC cells with CSC-like properties, CFE and stem cell markers expression do not guarantee the repopulation ability of the cells, when grafted into immune-deficient mice. In order to evaluate the tumorigenic ability of cSCC subpopulations, we grafted RAD and NRAD cells onto NOD-SCID mice, together with primary human fibroblasts, and monitored tumor growth over time. Given the strong dependence of skin tumor cells from the tumor microenvironment, the xenograft model employed in this study satisfies the requirements for the propagation of skin tumors *in vivo* [3,30]. RAD cells generated tumors 2–4 times bigger (in 100% of mice) than those formed by NRAD keratinocytes (in 95% of mice) (Figure 6A,B). The ability of RAD cells to form bigger tumors suggests that the RAD population is enriched either in tumor-initiating cells or rapidly growing/aggressive SCC keratinocytes, as compared to NRAD, thus retaining greater tumorigenic potential. However, since NRAD cells possess tumorigenic ability, albeit developing smaller tumors, high-β_1_-integrin expression is not an exclusive requirement for the selection of tumorigenic cells in cSCC. Hematoxilin and eosin staining confirmed that RAD-derived tumors are more aggressive and invasive than NRAD-derived ones. Pan-cytokeratin staining of RAD and NRAD derived tumors confirms the presence of epithelial cells in both tumor types (Figure 6C). While the tumorigenic ability of SCC cells separated by β_1_-integrin levels through collagen IV adhesion had not been previously shown, β_1_-integrin is considered as a key player in cSCC tumorigenesis. In fact, β_1_-integrin blockade reduces tumorigenesis *in vivo* [35], while mutations in the β_1_-integrin gene lead to malignant conversion of skin tumors [36]. This study only evaluated the tumorigenic ability of RAD and NRAD cells without analyzing their self-renewal ability through serial transplantation experiments. Therefore, we cannot address whether β_1_-integrin is a marker of CSCs in cSCCs.

### 2.7. RAD-Derived Tumors Display Most Aggressive Features

**Figure 7:**
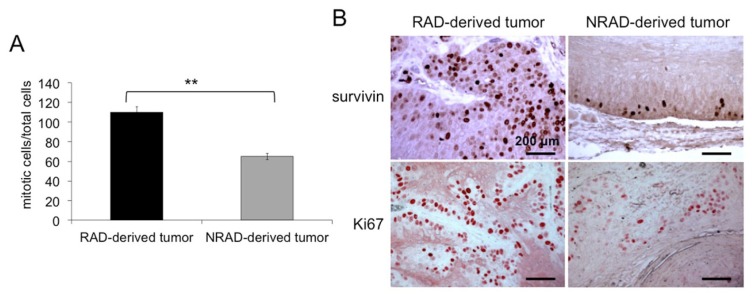

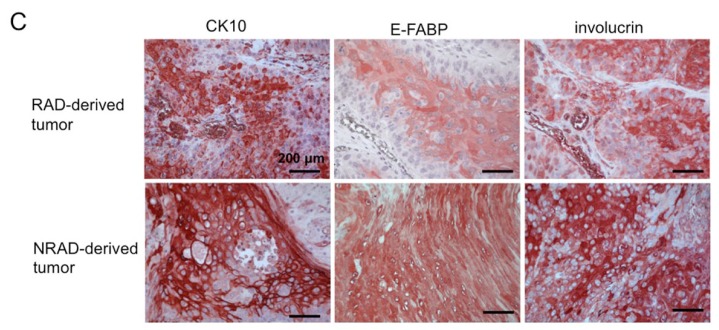
RAD and NRAD-derived tumor characterization. (**A**) Mitotic Index representing the number of cells undergoing mitosis over total cells were counted in RAD and NRAD-derived tumors **p < 0.01; (**B**) Survivin and Ki67 expression in RAD and NRAD-derived tumors by immunohistochemistry. Scale bar = 200 μm; (**C**) K10, E-FABP and involucrin expression in RAD and NRAD-derived tumors by immunohistochemistry. Scale bar = 200 μm.

To further characterize RAD-derived tumors, we calculated the mitotic index (MI) in RAD and NRAD-derived tumors. MI is often associated with tumor histological grades and cell proliferation since it predicts patient survival and resistance to therapies in many cancerous tissues. In RAD-derived tumors, MI was 2,68 fold higher than in NRAD derived lesions (RAD: 8.82+/−1.56, NRAD 3.29+/−0.97) (Figure 7A). Sections were also stained with survivin and Ki67 to evaluate cell proliferation. As previously reported, survivin is mainly localized in the nuclei of cancer cells, while it almost exclusively localizes in the cytoplasm of normal epithelial cells [7,37]. Cells with either survivin or Ki67 nuclear staining were more numerous in RAD than in NRAD tumors (Figure 7B). Ki67 overexpression in RAD-derived tumors is consistent with increased MI, while survivin increase in RAD tumors is in line with its role in cell cycle regulation and cell division [38]. The concomitant overexpression of survivin and Ki67 along with the high MI in RAD-derived tumors strongly suggests the superior aggressiveness of RAD-derived tumors. Survivin is more pronounced in less differentiated cSCC cells (manuscript in preparation). In addition, survivin is mainly localized in suprabasal epidermal layers in RAD tumors, while remaining in basal and immediately suprabasal cells in NRAD tumors. Along this line, the differentiation markers K10, E-FABP and involucrin were more expressed in NRAD than in RAD-derived tumors (Figure 7C).

All together, these findings suggest that RAD cells are poorly differentiated, highly proliferating and tumorigenic cSCC cells that in turn, generate very aggressive tumors. This seems to suggest that cells expressing high levels of β_1_-integrin and stem cell features determine higher tumor aggressiveness in cSCC.

## 3. Experimental Section

### 3.1. Isolation of Primary Keratinocytes from cSCCs Tissues

Tumor samples from ten human cSCC patients were surgically removed and immediately stored in a sterile test tube containing medium and antibiotics. All tumor samples were collected with written informed consent of patients, according to the Declaration of Helsinki after approval of the Modena Medical Ethical Committee. Tumor tissues were washed with PBS without calcium and magnesium, cut into small fragments and digested in DMEM containing 200 U/mL type I collagenase, 200 U/mL dispase and 70 U/mL DNase shaking for 2 h at 37 °C. The digested top tissue mixture was then filtered and centrifuged to collect the cells. Total cells were then seeded onto 3T3 feeder layers as previously described [39] and primary and secondary cell cultures were obtained.

For RAD and NRAD cells isolation, collagen IV coated plates were prepared by seeding a human placenta-derived collagen IV solution (100 μg/mL, Sigma-Aldrich, St. Louis, MO, USA). Total cells from cSCC cultures, either at passage 0 or 1, were seeded on collagen IV pre-coated dishes for 5 min. Cells adhering within 5 min represent RAD cSCC keratinocytes; in order to analyze RAD and NRAD cellular morphology, after cell separation, NRAD and TOT cells were collected and seeded on a dish of the same size as the one used for the separation; pictures have been taken within 10 min from the separation process. In order to avoid changes in the expression pattern due to prolonged cell cultures, immediately after separation NRAD cells were collected, while RAD cells were detached by incubating them with a trypsin/EDTA 0.05%/0.02% solution for 5–10 min at 37 °C. For *in vitro* proliferation/viability assays, cells were maintained in culture with serum-free keratinocyte growth medium (KGM) until they reached the desired confluency.

### 3.2. Western Blotting

Total proteins were extracted with RIPA lysis buffer containing protease inhibitors. Equal amounts of protein from each sample were run through a 6%–18% SDS–PAGE gel and transferred onto a nitrocellulose membrane. Membranes containing protein were incubated overnight at 4 °C with the following antibodies: anti-survivin rabbit polyclonal (1:1000; Novus Biologicals, Bloomington, MN, USA), or goat anti-human FABP5 (1:1000; R&D Systems, Inc. Minneapolis, MN, USA), or mouse mAB anti-human involucrin (1:6000; Sigma-Aldrich, St. Louis, MO, USA), or anti-human β_1_-integrin (1:500; Santa Cruz Biotechnology, inc. Santa Cruz, CA, USA) or anti-human β-actin (1:5000;

Sigma-Aldrich, St. Louis, MO, USA). After 3 washes with a PBS/tween solution, membranes were then incubated with secondary antibodies for 45 min at room temperature. Bands were then visualized with chemiluminescence detection system (Amersham Biosciences UK Limited, Little Chalfont Buckinghamshire, UK).

### 3.3. Detection of Cell Viability by Crystal Violet Staining

cSCC RAD, NRAD and bulk cells (5000/well) were seeded in a 96-wells plate and incubated at 37 °C for 72 h. Subsequently, the supernatants were discarded and the remaining viable adherent cells were fixed with 4% buffered paraformaldehyde and stained with a solution of 0.4% crystal violet in 100% methanol for 30 min. The plate was then rinsed with water and air-dried. The absorbance of each well was measured at 590 nm with a microplate reader. The results are expressed as optical density units (OD). Results are calculated as the mean SD of three different experiments. The OD of the samples was plotted against time.

### 3.4. BrdU Proliferation Assay

The BrdU proliferation assay for RAD and NRAD cells from cSCCs cultures was carried out using the FITC BrdU Flow Kit (BD Biosciences, San Jose, CA, USA). Isolated cells were re-seeded and incubated with Bromodeoxyuridine (BrdU, 1 mM) for 24 h. BrdU incorporating cells (S phase) were analyzed 72 h after the seeding following manufacturer’s instructions. Briefly, cells were fixed and permeabilized by BD Cytofix/Cytoperm Buffer. After 40 min incubation with DNase at 37 °C, cells were stained with FITC-conjugated anti-BrdU monoclonal antibody. 7-aminoactinomycin (7-AAD) was added to each sample right before flow Cytometry analysis (Epics XL flow cytometer (Coulter Electronics Inc., Hi-aleah, FL, Beckman Coulter, Fullerton, CA, USA, http://www.beckmancoulter.com). The results are expressed as the % of cells in each phase of the cell cycle and are calculated as the mean SD of three different experiments.

### 3.5. Colony Forming Efficiency (CFE)

Keratinocytes were cultured on a feeder layer composed of mytomicin C (Sigma-Aldrich, St. Louis, MO, USA)-treated 3T3 cells at a density of 100 cells per dish. Fourteen days later, dishes were fixed with 10% buffered formalin and stained with crystal violet. Colonies that contained more than 50 cells were counted and CFE was calculated. The colony number was expressed as a percentage of the number of cells plated in each dish. Results are calculated as the mean ± SD of three different experiments.

### 3.6. Scratching Assay

cSCC keratinocytes were seeded in dishes (25 × 10^3^ cells/cm^2^) and treated with 5 μg/mL of mitomycin C (Sigma-Aldrich, St. Louis, MO, USA) for 2 h. After 2 washes with sterile PBS, each well was then drawn along the cell monolayer with a sterile plastic tip. Plates were then washed twice with PBS and incubated in serum-free medium. Cells were then monitored at 48 h. The result of each experiment was expressed as the % of the mean of migrated cells from six different areas. The final results are expressed as the mean ± SD of three different experiments. Student’s *t*-test was performed for comparison of the means.

### 3.7. H&E Staining and Mitotic Index Calculation

Tumors were removed from animals, placed in 4% buffered formalin for no more than 24 h, and then embedded in paraffin. Serial tissue sections (4 μm thick) were deparaffinized, hydrated in xylene and graded alcohol solutions. For H&E stains, Mayer hematoxylin and Eosin Y Alcoholic were used. Staining times were 5 min for hematoxylin and 1 min for eosin. Slides were covered with coverslips with the addition of aqueous mount. The mitotic index was calculated and expressed as the ratio between the number of cells in mitosis and the total number of cells on H&E sections.

### 3.8. *In Vivo* Tumorigenesis

cSCC tumors were generated as previously shown [40]. Briefly, cSCC subpopulations were separated from SCC13 keratinocytes. 1–5 × 10^6^ SCC13 keratinocytes were immediately mixed with 1 × 10^6^ neonatal dermal fibroblasts, and injected into silicon chambers (Renner GMBH, Darmstadt, Germany) surgically implanted on the dorsal fascia of recipient NOD/SCID mice. The upper part of the chamber was removed one week after injection, in order to allow air exposure of the graft. At day 14 from implantation, the chamber was entirely removed. Reconstituted skin tumors were typically observed within 1–3 months post-grafting. Tumor growth was monitored twice/week and measured with a caliper. Tumors were harvested when they reached 1 cm diameter size for histological analysis, mitotic index measure and immunohystochemistry. Tumor volume was calculated by the formula (longest diameter) × (shortest diameter)^2^/2.

### 3.9. Immunohistochemistry

Tumor sections were obtained from formalin fixed-paraffin embedded RAD and NRAD-derived tumors. The staining was performed using the UltraVision LP Detection System AP Polymer & Fast Red Chromogen assay (Thermo Fisher Scientific, Waltham, MA, USA), according to the manufacturer’s instructions. Briefly, slides were treated with Ultra V Block and samples were incubated with anti-CK10 (1:200; Epitomics, Burlingame, CA, USA) or anti-E-FABP (1:200; R&D systems, Minneapolis, MN, USA), or anti-Ki67 (1:200; Epitomics, Burlingame, CA, USA), or anti-keratin (ready to use; Cell Marque), or anti-involucrin (1:500; Sigma-Aldrich, St. Louis, MO, USA) for 1 hat room temperature. After washes in PBS, Primary Antibody Enhancer (Thermo Fisher Scientific, Waltham, MA, USA) was added for 20 min at room temperature, followed by incubation with AP Polymer anti-mouse/rabbit IgG for 30 min at room temperature. Slides were stained with Fast Red using Naphthol Phosphate as substrate. Samples were analyzed under a conventional optical microscope (Zeiss Axioskope 40, Carl Zeiss, Jena, Germany).

For survivin detection, the slides were first boiled in citrate buffer, pH6, for 30 min at 98 °C. samples were then incubated with anti- survivin rabbit polyclonal antibody (1:300; Novus, Bloomington, MN, USA) for 32 min at 37 °C. For immunohistochemistry staining, we used 3,3′ Diaminobenzidine (DAB) as cromogen.

### 3.10. Real Time PCR

Total RNA was extracted from primary RAD and NRAD cSCC keratinocytes by using TRI Reagent, performed as described by the manufacturer (Sigma-Aldrich, St. Louis, MO, USA). cDNA was synthesized from 500 ng of total RNA as described by the manufacturer (Roche). Each cDNA generated was amplified by quantitative Real Time PCR in a reaction mixture containing 1× iQ™ SYBR^®^ Green Supermix (Bio-Rad Laboratories, Hercules, CA, USA), 10 ng of cDNA, and the following primers: Oct-4: 5′-AGCAAAACCCGGAGGAGT-3′ and 5′-CCACATCGGCCTGTGTATATC-3′, Sox-2: 5′-TTGCTGCCTCTTTAAGACTAGGA-3′ and 5′-CTGGGGCTCAAACTTCTCTC-3′, Nanog: 5′-TGAACCTCAGCTACAAACAG-3′ and 5′-TGGTGGTAGGAAGAGTAAAG-3′, GAPDH: 5′-AGCCACATCGCTCAGACA-3′ and 5′-GCCCAATACGACCAAATCC-3′. The expression of each gene was calculated by the comparative threshold cycle method (2^−ΔΔ^*^C^*^t^). Briefly, the average *C*t value of GAPDH for every sample was subtracted from the *C*t value for each target gene, resulting in the Δ*C*t value. The expression level of each gene is shown relative to that of RAD cells (calibrator). The comparative cycle threshold (ΔΔ*C*t) results from the difference between (Δ*C*t each population) minus (Δ*C*t calibrator). PCR was carried out at least three times for each sample, and the experiment was performed at least three times.

## 4. Conclusions

Here we show that tumor cells isolated from primary cSCC based on their highest β_1_-integrin expression (RAD) have stem cell features. *In vivo*, RAD cells generate large and aggressive tumors characterized by elevated mitotic index and hyperproliferation. As nuclear survivin, a KSC marker, is abundantly expressed in RAD-derived tumors, we speculate that survivin plays an important role in the development of cSCC, thus confirming that this tumor is derived from KSC. Functional studies are needed not only to fully understand the role of survivin in tumor formation, but also to determine whether survivin, similarly to β_1_-integrin, is involved in CSC activities, being ultimately responsible for tumor recurrence and metastasis in cSCC.
